# Influence of pre and postpartum alpha-tocopherol supplementation on milk yield, milk quality, and udder health of Jersey crossbred cows at tropical lower Gangetic region

**DOI:** 10.14202/vetworld.2020.2006-2011

**Published:** 2020-09-26

**Authors:** Amit Kumar Singh, Champak Bhakat, Tripti Kumari, Dilip Kumar Mandal, Anupam Chatterjee, Muthupalani Karunakaran, Tapas Kumar Dutta

**Affiliations:** 1 Livestock Production Management Section, ICAR-National Dairy Research Institute, Eastern Regional Station, Kalyani, West Bengal, India; 2 Animal Nutrition Section, ICAR-National Dairy Research Institute, Eastern Regional Station, Kalyani, West Bengal, India; 3 Animal Reproduction Section, ICAR-National Dairy Research Institute, Eastern Regional Station, Kalyani, West Bengal, India

**Keywords:** alpha-tocopherol, hot-humid region, jersey crossbred, milk production, udder health

## Abstract

**Background and Aim:**

Alpha-tocopherol supplementation influences milk yield, milk quality, and udder health of dairy cows, which needs to be investigated for Jersey crossbred cows at hot-humid climate. Therefore, the present study was framed with an objective to study the effect of pre and postpartum Alpha-tocopherol supplementation on milk yield, milk quality, and udder health status of Jersey crossbred cows at tropical climate.

**Materials and Methods:**

For this study, 19 similar parity, body condition score, and production level dairy animals were separated randomly into three groups, namely, Control, T1 and T2. Control group (no supplementation) was compared with two treatment groups, namely, T1 and T2. Supplementation of Alpha-tocopherol was done in concentrate fed to the animals (at 1 g/cow/day) 30 days prepartum to 30 days postpartum in T1 and 30 days prepartum to 60 days postpartum in T2 groups. Observations were taken for different parameters up to 5 months of lactation.

**Results:**

Statistically analyzed data revealed that overall significantly (p<0.01) more milk production was found in T2, followed by T1 than the control group. Overall significantly (p<0.01) lower somatic cell counts and modified California mastitis tests were recorded in T2, followed by T1 than the control group. Overall significantly (p<0.01), better milk quality in terms of methylene blue reduction test was found in T2, followed by T1 than control groups. Differences in the milk composition of all three groups were non-significant (p>0.05).

**Conclusion:**

Supplementation of Alpha-tocopherol during prepartum to initial lactation period may enhance milk yield, milk quality, and udder health status of Jersey crossbred cows at the tropical lower Gangetic region.

## Introduction

Improvement in milk yield, milk quality, body condition, and udder health status can be achieved with proper nutrition and management conditions during the initial and later lactation period [1,2]. Adoption of good animal management may improve the overall production and health performance of dairy cows in tropical climate [3-5]. However, due to poor availability of good quality fodder and adverse climatic conditions, it becomes difficult to maintain proper nutrition and management conditions for dairy animals, the farmers of tropical climate [6]. An approach for Alpha-tocopherol supplementation to dairy cows during the pre and postpartum period has the ability to improve postpartum milk production, udder health, and body condition performance [7]. Farmers may adapt this approach for healthier milk production with the better body conditions of dairy cows in the tropical climate.

Subclinical mastitis (SCM) in Jersey crossbred cows of dairy owners is one of the most challenging tasks in tropical regions as it leads to high production, health, and economic losses to the dairy animals [8]. SCM is one of the important reasons which affect postpartum performance of dairy animals and finally, leading to lower lactation yields [9], inferior milk quality, and SCM is ranked first among different ailments which cause considerably high loss to the dairy owners. It is a global problem which adversely affects animal health, milk quantity, and milk quality [10], and every country, including developed ones also, suffers huge financial losses. Particularly for the backyard farmers in the developing world, SCM is one of the most economically devastating problems [6]. Increased milk demand has given emphasis to crossbred animals, but researches have shown that crossbred animals are more prone to the risk of intra-mammary infection (IMI) [11]. Hence, proper management practices should be adopted to avoid such losses to the dairy farmers.

Alpha-tocopherol (Vitamin-E) is an important membrane antioxidant that enhances the functional efficiency of neutrophils by protecting them from oxidative damage, followed by the intracellular killing of ingested bacteria [12] and keratinization of the teat canal in lactating animals [13]. Vitamin E is an essential fat-soluble vitamin, and it is not synthesized in the rumen. Therefore, supplementation of Vitamin E must be done to meet the requirement of dairy cows [14], especially in the case when the quality of fodder available is poor. Tropical climatic conditions offer harsh climatic conditions for the production of good quality fodder [6]. Vitamin E supplementation has been seen to consistently improve the neutrophil function in dairy cows [15,16]. Supplementation of Vitamin E during the dry period has been observed to reduce the incidence of mastitis cases in postpartum period in cows by potentiating the natural defense of the mammary gland through increased serum Alpha-tocopherol concentrations [17]. Its supplementation may be beneficial in early lactation as there is an increased risk of SCM in the early lactation period in dairy animals [18].

Studies on supplementation of Vitamin E are mostly either during the prepartum or postpartum period, but no confirmative studies are present on supplementation of Vitamin E during both pre and postpartum periods at the hot-humid climate for Jersey crossbred cows. Therefore, the present study was aimed to study the effect of pre and postpartum Alpha-tocopherol supplementation on milk yield, milk quality, and body condition of Jersey crossbred cows at hot-humid climate.

## Materials and Methods

### Ethical approval and informed consent

Ethical approval is not necessary for such type of study. However, this study complies with ethics, and all the animals were properly reared by their respective owners. No animals were harmed during this study. Informed consent was obtained from all the participants.

### Study area

The present study was carried out from September 2018 to June 2019 at Eastern Regional Station-National Dairy Research Institute (ERS-NDRI), institute’s adopted village (Muratipur) of Nadia district, West Bengal, which has hot-humid climate. In the village, an infrastructure has been established at this village named Dairy Vikas Kendra of ERS-NDRI from where the records of animals’ parity, previous production, Artificial Insemination date, and expected date of calving were collected for selecting suitable animals for this experiment. The latitude and longitude position being 22°56′30″N and 88°32′04″E, respectively.

### Methodology for field experimentation of Alpha-tocopherol

The methodology followed for field experimentation of Alpha-tocopherol was as follows:


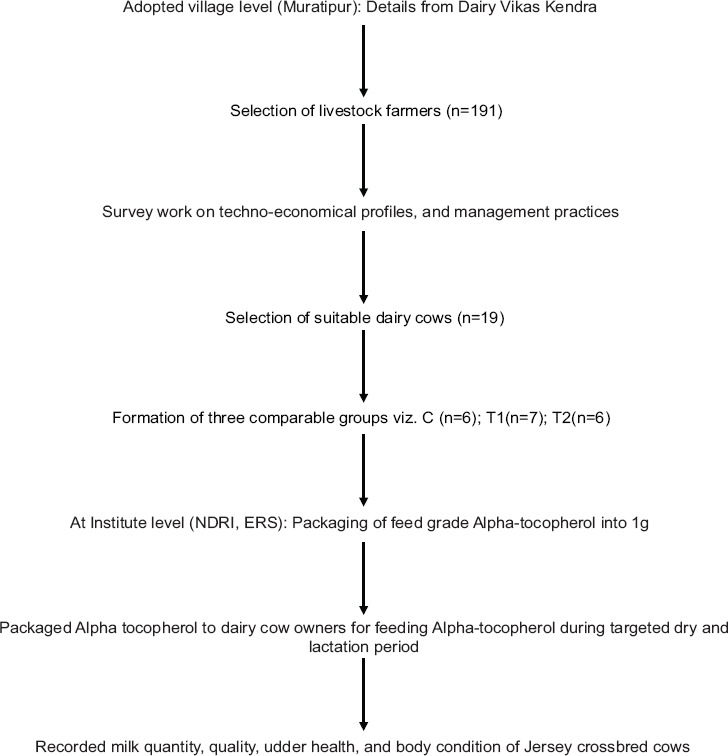


#### Selection of livestock farmers (n=191)

In Muratipur village of Nadia district, 191 respondents were surveyed who were keeping dairy cows for their livelihood. Most of them were marginal or small farmers with two or three dairy animals. Respondents were surveyed for udder health management practices and management practices followed by dairy farmers. The survey indicated that poor udder health management practices were followed by almost all the farmers.

#### Selection of suitable dairy cows (n=19)

Out of 191 surveyed respondents, dairy cows (n=19) of different farmers which were in the last trimester of gestation were selected for field experimentation based on their parity (3-4), the mean level of production (7.8-8.0 kg/day/cow in each group), and initial body condition score (BCS) (3.38-3.43). Keeping in mind, the poor economic background of dairy farmers, Alpha-tocopherol of feed grade was procured (Rs. 1.4/g) for supplementing at 1 g/d/cow to those selected cows from 30 days pre to 60 days postpartum period in two different treatment groups than the control group.

### Involvement of research assistants

Competent and trained research assistants used to supply the pre-packaged 1 g Alpha-tocopherol to the selected dairy cows during the targeted days of dry as well as lactation period to feed them. Following which the udder health and other parameters were measured in the NDRI- ERS, Livestock Production Management section laboratory under aseptic conditions, and BCS were measured on a fortnight basis. However, these fortnight evaluations were utilized for parameters on a monthly basis.

### Experimental animals

This experimentation was conducted on 19 Jersey crossbred cows having similar initial BCS, body weight, and parity and observed during the dry period (30 days pre-calving), during calving and postpartum up to 5 months of the lactation period. Based on BCS, parity, level of production in the previous lactation, and body weight during drying off time (after completion of full lactation), animals were randomly divided into three different comparable groups, that is, Group-1 as T1 and Group-2 as T2 and control group. T1 comprised seven animals supplemented with Alpha-tocopherol at 1g/cow/ day for 30 days prepartum and 30 days postpartum period; in T2, six cows were supplemented with Alpha-tocopherol at 1 g/cow/day for 30 days prepartum and 60 days postpartum period, and in control group, traditional feeding management was done without Alpha-tocopherol supplementation.

All groups of animals were provided (traditional feeding management practice) with good quality *ad libitum* green and dry fodders during the dry period. During postpartum period provided with a concentrate mixture (at 3 kg/cow/day) along with *ad libitum* good quality green and dry fodders. The DCP and TDN contents of the concentrate mixture were 14% and 68%, respectively. However, animals were apparently healthy without any kind of disorder. Housing and other management practices were similar to all groups. Animals were stall-fed with the provision of separate manger and watering facilities [19].

### Analysis of milk samples

About 70 ml of composite milk samples were collected from every animal after thoroughly mixing whole milk bucket after the milking process is completed of individual animals in sterilized glass bottles at fortnight intervals. Udder health status (SCM) was detected by somatic cell count (SCC) and modified California mastitis test (MCMT) in the milk sample. The MCMT was conducted as per the procedure adopted by Bharti *et al*. [20]. Methylene blue reduction test (MBRT) was performed to assess the quality aspect of milk samples.

### SCC in milk

SCC in milk samples was analyzed by an automatic machine of DeLaval cell counter DCC. The microscopic procedure [11] for cross-checking of SCC was also followed by the microscopic method of SCC using modified Newman’s Lampert stain and then examined under the oil immersion lens (100×). A total of thirty different fields per smear per slide were accessed, and mean the number of cells per field was then multiplied by a microscopic factor to obtain the total number of cells present in per ml of milk.

### Milk quality test

MBRT was carried out to predict the milk quality in this study. In MBRT, the color provided to milk samples by the addition of methylene blue dye happens to disappear more or less quickly based on bacterial load in the milk sample. Thus, the time taken for the reduction of imparted color was considered to measure of the number of microorganisms in the milk sample [7].

### Statistical analysis

SPSS statistics 21.0 software (IBM Corp., NY, USA) was utilized for meticulous statistical analysis of each and every parameter. The univariate General Linear Model method was followed for analysis of variance in different parameters. It was followed by comparisons of means for all pairs using Duncan’s New Multiple Range Test. Pearson’s correlation method was utilized for determining the correlation coefficients. The level of significant differences was checked at p<0.05 and p<0.01.

## Results and Discussion

### Milk yield and milk composition

Statistically analyzed data (Table-1) revealed that significantly (p<0.01) higher overall milk yield was observed in T2, followed by T1 than the control group. A similar trend of observation was recorded every month for up to 5 months of the lactation period. The month-wise variation in milk production indicated that T2 group reached their peak production during 3^rd^ month, whereas T1 and control group reached their peak production 2^nd^ month. Comparatively higher persistency of peak milk yield was recorded in treatment than control group. Highest milk was recorded in T2 (10.66 kg/d/cow), followed by T1 (10.31 kg/d/cow) and control group (9.70 kg/d/cow). Significantly (p<0.01), higher total milk yield was found in T2 followed by T1 than the control group in each month of the lactation period under this study. The reason of significantly (p<0.01) higher milk production in the treatment group might be due to Alpha-tocopherol (Vitamin E) supplementation during pre and post-calving period, which seemed to help in reducing the SCM cases which occurs commonly during 1^st^ week of lactation, through improved antioxidant function and keratinization of the teat canal. Garai *et al*. [21] showed that Jersey crossbred cow of this tropical region may have milk production of 9.60 kg/day/cow. However, in a farm experimentation, Singh *et al*. [1] found that Jersey crossbred cows of this region may produce 12.70 kg/day/cow under organized conditions. Duffield *et al*. [22] remarked that high negative energy balance was found to be associated with milk yield. Vitamin E supplementation has been found to reduce the cases of SCM [12]. The results of this study are in line with the findings of Chatterjee *et al*. [23]. However, LeBlanc *et al*. [24] found contradictory results which indicated that supplementation of Vitamin E had no effect on udder health.

**Table-1:** LSQ mean±SE of milk yield and milk composition of Jersey crossbred cows.

Months	Control (n=6)	T1 (n=7)	T2 (n=6)
Milk yield (kg/day/cow)	7.88^a^±0.08	9.19^b^±0.04	9.68^c^±0.09
^1^Fat	4.70±0.08	4.83±0.04	4.85±0.05
^1^Total SNF	8.60±0.04	8.73±0.03	8.74±0.04
^1^TS	13.29±0.08	13.56±0.05	13.59±0.06
^1^Protein	3.81±0.03	3.82±0.04	3.82±0.05

Means bearing different superscript differ significantly (p<0.01) column wise.

1 (%/day/animal); Total SNF=Total solid not fat, TS=Total solids, n=Number of animals in each group

Statistically analyzed data (Table-1) revealed that there was a non-significant difference of fat, solid not fat (SNF), total solid, total protein of control, and T1 and T2 group’s milk samples. A similar trend of composition parameters was recorded in all 5 months of the lactation period among the three groups. However, milk samples of all groups contained normal and standard amounts of total fat, total SNF, total solid, and total protein. The total fat %/day/cow was found to be numerically higher in T2, followed by T1 than the control group. A similar trend of observation was recorded for total SNF, total solid, and total protein %/day/cow among 3 groups. Bharti *et al*. [20] reported that IMI and the incidence of SCM caused considerable changes in milk SCC, milk yield, and pH of the milk. Milk samples obtained from SCM infected animals had lower test day milk yield, fat%, and SNF% but higher SCC and milk pH. It has been stated that doses higher than 9000 IU of Vitamin E per day, in diets may increase the fat content of cow’s milk during the lactation period [25]. Prepartum immune suppression was multifactorial but was associated with endocrine changes and decreased intake of critical nutrients. Smith *et al*. [26] reported that dry cows fed approximately 1000 IU of supplemental Vitamin E/day during the 60 days dry period and (or) injected with approximately 50 mg of selenium 21 days before expected calving had fewer cases of clinical mastitis with reduced duration compared with cows not fed Vitamin E or injected with selenium.

### Milk quality

Statistically analyzed data (Table-2) revealed that month-wise overall significantly (p<0.01) lower MBRT values were found in control than T1 followed by T2 groups. A similar trend of MBRT was recorded in all 5 months of the lactation period among the three groups. This might be due to the fact that color, which was provided to milk by the addition of a dye such as methylene blue, gets disappeared more or less quickly depending on the microorganism load in the milk sample. Removal of oxygen from milk samples and the formation of reducing substances during bacterial metabolism cause the disappearance of color. Bacteria present in milk samples were responsible agents for oxygen consumption, which were more in the number of control group samples than treatment groups. Kumari *et al*. [27] showed that supplementation of trisodium citrate was also an easy and economic management practice to increase milk yield and milk quality. Bhakat *et al*. [11,28] reported that the IMI leads to changes in the glandular tissue of the udder. Hence, it is essential to monitor IMI in dairy cows to maintain milk quantity, quality, and udder health. Paul and Bhakat [29] had investigated that higher SCC due to poor management practices was very critical because more influx of milk SCC, which not only disrupts the mammary epithelium but also found to decrease the milk quality, which ultimately leads to the lower returns. Supplementation of Vitamin E positively affects the functioning of neutrophils and milk quality in the organized herd, and 4000 IU Vitamin E supplementation during the past 14 days of the dry period was found to improve udder health [15].

**Table-2:** LSQ mean±SE of MBRT (min) for milk quality test at tropical lower Gangetic region.

Months	Control (n=6)	T1 (n=7)	T2 (n=6)
1	97.50^a^±11.60	138.21^b^±16.13	213.75^c^±22.47
2	80.00^a^±16.41	133.93^b^±15.28	225.00^c^±16.42
3	81.25^a^±13.42	135.00^b^±13.25	200.00^c^±13.15
4	78.75^a^±11.33	131.79^b^±18.29	202.50^c^±19.45
5	81.25^a^±16.25	150.00^b^±10.23	197.50^c^±21.45
Overall	83.75^a^±13.80	137.79^b^±14.64	207.75^c^±18.59

Means with different superscripts differ significantly (p<0.01) from each other row wise. n=Number of animals in each group, MBRT=Methylene blue reduction test

### Udder health status

#### SCC (cells/ml of milk)

Statistically analyzed data (Table-3) revealed that overall significantly (p<0.01) lower SCC (cells/ml of milk) were found in T2 and T1 than the control group. Non-significant differences were found between T1 and T2 groups. After the retransformation of Log_10_ SCC was done, milk samples of the cow of treatment groups (T2 and T1) showed normal SCC, which was generally required for the self-defense mechanism of udder, while milk samples of cow of the control group showed SCC more than 200,000 in all most all months of lactation period which was the clear indication for the occurrence of SCM, in which visible symptoms were not found but milk yield became lesser and gradually lesser with the passage of lactation period in this study. The coefficients of correlation (−0.449) indicated a negative and significant (p<0.01) correlation between SCC and milk production of this study. SCC, MCMT, and surf filled mastitis tests were appropriate testing for SCM in dairy cows, as suggested by Kumari *et al*. [27]. Bharti *et al*. [30] observed that delay in post milking feeding time may lead to IMI and SCM in Jersey crossbred cows under tropical climate. Bhakat *et al*. [31] reported that the Log_10_ SCC (cells/ml) were significantly (p<0.01) more in IMI cows (6.55±0.05) as compared to non-IMI Jersey crossbred cows (4.05±0.04).

**Table-3:** Milk testing parameters for udder health/subclinical mastitis in Jersey crossbred cows.

Months	Control (n=6)	T1 (n=7)	T2 (n=6)
Log_10_SCC (cells/ml)			
1	5.86^a^±0.02	5.10^b^±0.04	5.05^b^±0.02
2	5.93^a^±0.03	4.96^b^±0.02	5.00^b^±0.03
3	5.94^a^±0.01	4.96^b^±0.03	4.97^b^±0.04
4	6.01^a^±0.05	5.02^b^±0.01	4.99^b^±0.02
5	6.04^a^±0.04	5.02^b^±0.02	4.89^b^±0.03
Overall	5.96^a^±0.03	5.01^b^±0.02	4.98^b^±0.03
MCMT (grades)			
1	3.42^a^±0.19	2.00^b^±0.13	1.58^c^±0.15
2	3.58^a^±0.16	1.79^b^±0.15	1.58^b^±0.19
3	3.67^a^±0.21	1.71^b^±0.14	1.58^b^±0.18
4	3.67^a^±0.18	2.07^b^±0.11	1.92^b^±0.12
5	3.83^a^±0.17	1.86^b^±0.10	2.00^b^±0.14
Overall	3.63^a^±0.18	1.89^b^±0.13	1.73^b^±0.16

Means with different superscripts differ significantly (p<0.01) from each other row wise. n=Number of animals in each group. MCMT=Modified California mastitis test

### MCMT

MCMT of milk samples showed higher grade value in control than treatment groups of cows, which represented poor quality of milk in the control group. Statistically analyzed data (Table-3) expressed that there was lesser MCMT value for treatment (T1 and T2) than control groups in every month of the observed lactation period. Significantly (p<0.01) more MCMT values were found in control than T1 and T2 groups. However, non-significant differences (p>0.05) were studied between T1 and T2 groups in all most all months except 1^st^ month. This might be due to the presence of higher SCC leading to the increased alkalinity of milk. The presence of more DNA by virtue of increased somatic reacts with MCMT reagents, which are found to increase the grades of MCMT in control than treatment. The coefficients of correlation (−0.407) indicated a negative and significant (p<0.01) correlation between MCMT and milk yield of this study. Bhakat *et al*. [6] reported that through machine milking practice, IMI and MCMT can be reduced as compared to hand milking practices with higher milk yield and milk quality in Jersey crossbred cows at tropical climate.

## Conclusion

Poor milk yield, milk qualities with the poor udder health status of dairy cows are important problems to be worked at tropical climate. There is a potential to overcome these silent problems through the supplementation of Alpha-tocopherol. From this study, it can be concluded that Alpha-tocopherol supplementation (at 1 g/cow/day) 30 days prepartum to 60 days postpartum may improve postpartum performance followed by Alpha-tocopherol supplementation (at 1 g/cow/day) 30 days prepartum to 30 days postpartum as compared to non-supplemented group by improved milk yield, milk quality, and improved udder health status of Jersey crossbred cows at the tropical region.

## Authors’ Contributions

AKS and CB designed, conceptualized, and wrote the manuscript of this study. TK assisted in laboratory testing. DKM assisted in data interpretation. AC helped in structuring the manuscript. MK and TKD had helped in animal selection and writing manuscript. All authors read and approved the final manuscript.
